# A Review on Manufacturing and Post-Processing Technology of Vascular Stents

**DOI:** 10.3390/mi13010140

**Published:** 2022-01-16

**Authors:** Wei Jiang, Wenxiang Zhao, Tianfeng Zhou, Liang Wang, Tianyang Qiu

**Affiliations:** 1 School of Mechanical Engineering, Beijing Institute of Technology, No. 5 Zhongguancun South Street, Haidian District, Beijing 100081, China; jiangv@bit.edu.cn (W.J.); liangwangalex@outlook.com (L.W.); 2 Key Laboratory of Fundamental Science for Advanced Machining, Beijing Institute of Technology, No. 5 Zhongguancun South Street, Haidian District, Beijing 100081, China; zhaowx@bit.edu.cn (W.Z.); zhoutf@bit.edu.cn (T.Z.)

**Keywords:** vascular stents, manufacture, post-processing, machining quality, biocompatibility

## Abstract

Percutaneous coronary intervention (PCI) with stent implantation is one of the most effective treatments for cardiovascular diseases (CVDs). However, there are still many complications after stent implantation. As a medical device with a complex structure and small size, the manufacture and post-processing technology greatly impact the mechanical and medical performances of stents. In this paper, the development history, material, manufacturing method, and post-processing technology of vascular stents are introduced. In particular, this paper focuses on the existing manufacturing technology and post-processing technology of vascular stents and the impact of these technologies on stent performance is described and discussed. Moreover, the future development of vascular stent manufacturing technology will be prospected and proposed.

## 1. Introduction

Cardiovascular diseases (CVDs) are a leading killer of human life throughout the world, and most of these deaths are caused by atherosclerosis [1]. Atherosclerosis is made up of fat, cholesterol, calcium, and other substances inside the arteries that cause the blockage of blood vessels [2]. Percutaneous coronary intervention (PCI) with stent implantation is one of the most effective treatment to unblock blood vessels [3,4]. However, there are still many complications, including in-stent restenosis, late thrombosis, artery injury, re-occlusion rates, and local chronic inflammation [5], so that further research on vascular stent technology is urgently required.

Over the past few decades, vascular stents have experienced rapid development in terms of materials and design. In 1964, Charles et al. [6] used a catheter to expand a patient’s diseased blood vessels, which was the first percutaneous transluminal angioplasty (PTA). In 1969, Dotter et al. [7] carried out the first animal experiment of vascular stent implantation. In 1977, Andreas et al. [8] performed the first case of percutaneous coronary angioplasty (PCA), which opened a new era in the treatment of cardiovascular diseases. In 1985, Palmaz et al. [9] developed balloon-expandable stents to treat cardiovascular stenosis. In 1987, Sigwart et al. [10] successfully performed the world’s first coronary stent implantation operation.

The first generation of vascular stents were bare metal stents (BMSs), commonly made of stainless steel and nickel–titanium (NiTi) alloy. BMSs have many advantages including simple design, convenient processing, and excellent mechanical properties, which promote its wide use in the treatment of cardiovascular diseases and wide recognition by medical experts and patients [6,11,12]. However, BMSs will stay in the human body permanently after implantation, and clinical reports show that the BMSs will cause artery injury, inflammation, and even in-stent restenosis [13,14,15]. In order to solve these problems, the second generation of vascular stents, drug eluting stents (DESs), was developed [16]. DESs are the most widely used vascular stents in the current PCI treatment field. DESs include three parts: a metallic platform, an effective therapeutic agent, and a drug carrier. The metallic platform plays a role in supporting the blood vessels to maintain blood flow smoothly. The therapeutic agent and drug carrier form a drug coating to control the release of the therapeutic agent in blood vessels. Previous clinical studies have shown that DESs can effectively prevent inflammation of the diseased blood vessel and reduce intimal hyperplasia [17,18]. However, DESs have the same deficiency as BMSs due to the permanent retention in the human body [19]. Then, researchers developed the third generation of vascular stents, biodegradable stents (BDSs) [20,21]. BDSs are made of biodegradable materials including biodegradable metallic alloys and biodegradable polymers. BDSs can degrade and be absorbed after fulfilling their purpose of supporting diseased vascular. Therefore, BDSs have better biocompatibility and fewer complications. However, there are still some problems in the development of BDSs, especially weak mechanical properties and unclear degradation behavior [22,23,24], so that the long-term efficacy and safety of the BDSs are still under further research.

With the development of medical technology and the increasing demand for personalized patients, stent design optimization and precision manufacturing are highly required. At present, design optimization mainly focuses on the following aspects including bridge/link [25,26,27,28,29], representative volume element/representative unit cell [30,31,32,33,34], and patient-specific structure [35,36,37,38]. Then, the commonly used manufacturing technologies of vascular stents mainly include the braiding technique [39,40], micro-injection molding [41,42,43], laser cutting [44,45,46,47,48,49,50,51,52,53,54,55,56], and 3D printing [57,58,59,60,61,62,63,64]. Each processing method has its advantages and disadvantages. After stent processing, a series of post-processing techniques, including drug coating [65,66,67,68,69,70], surface modification [71,72,73,74], surface microstructures [75,76,77,78,79] can significantly improve the surface quality and the biocompatibility of the stent. Therefore, the efficacy and safety of vascular stents largely depend on precision manufacturing technology and post-processing technology.

This paper aimed to conclude the vascular stent technology in terms of stent materials, stent manufacturing techniques, and stent post-processing techniques. The existing stent manufacturing techniques are introduced, and the future manufacturing techniques are discussed. Finally, prospects and suggestions for the development of stent manufacturing technology will be determined.

## 2. Clinical Trials

Palmerini et al. [80] investigated the long-term safety of BMSs and DESs. The 3 year follow-up data of more than 50,000 patients showed DESs had great advantages in safety and efficacy over BMSs. Sousa et al. [81] evaluated the safety and efficacy of sirolimus (a cell cycle inhibitor)-coated BX Velocity stents, and the results showed that no major events had occurred during 8 months follow-up. Brugaletta et al. [82] compared the 1 year outcome among BMSs (Multilink Vision, Abbott Vascular, Santa Clara, CA, USA), everolimus-eluting stents (EESs) (Xience V, Abbott Vascular), bioresorbable vascular scaffold (BVS) (Abbott Vascular) in ST-segment elevation myocardial infarction patients. Many evaluations were conducted during the investigation including cardiac death, target vessel myocardial infarction, target lesion revascularization, and device thrombosis. Three kinds of stents showed similar performance at 1 year follow up.

A vascular stent is effective in the clinical treatment of vascular diseases, but there are still many problems including in-stent restenosis and stent thrombosis. After implantation, stent fracture seriously affects the performance of stent. The first report of stent fracture was a fracture on the midportion of a metal stent in 2002 [83]. Scheinert et al. [84] performed a follow-up survey on patients treated by implantation of self-expanding nitinol stents, and the results showed the rate of stent fractures was 37.2%, which indicates the risk of long-term implantation. Shaikh et al. [85] investigated stent performance in 3920 patients over 12 months, and 188 in-stent restenosis cases were observed. Stent fractures were identified in 35 of the 188 cases. Doi et al. [86] classified the stent fractures into four types and analyzed the fracture mechanisms. There are many stent fractures factors, including materials, design, and mechanical property. Therefore, the selection of appropriate materials and processing technology is crucial for the clinical application of vascular stents.

## 3. Stent Materials

### 3.1. Traditional Materials

Traditional vascular stents are commonly made of biomedical metals or alloys. For example, 316 L stainless steel has high mechanical strength and good corrosion resistance, and it is the original material used for vascular stents. However, 316 L stainless steel has poor flexibility, which may cause stent breakdown and medical complications. NiTi alloy was also used for vascular stents due to the fact of its good shape memory properties and excellent elasticity [87,88], while NiTi alloy stents brought out high internal stress when implanted in narrowed blood vessels. Then, cobalt alloy was used as vascular stent material, such as in the Wallstent stent (Boston Scientific, Marlborough, MA, USA). Cobalt alloy has good biocompatibility, corrosion resistance, and autoradiography properties. Moreover, the mechanical property of cobalt alloy is better than 316 L stainless steel, which contributes to thinner struts of cobalt alloy stents. Cobalt alloy stents with thinner struts generate less blood vessel coverage so that the reendothelialization of blood vessels can be accelerated and thrombosis can be reduced.

Although traditional metallic stents have achieved reasonable clinical outcomes, some shortcomings were also discovered after long-term clinical application. Traditional metallic stents stay in the human body permanently after implantation, which seriously affects the clinical prognosis of patients. In addition, a large number of complications will appear including in-stent restenosis, late thrombosis, artery injury, occlusion rates, and local chronic inflammation. It has been reported that the ratio of in-stent restenosis is 20–30% after the implantation of traditional vascular stents.

### 3.2. Biodegradable Materials

The biodegradable materials used for stents include corrodible metallic materials, such as magnesium alloys and zinc alloys, and degradable polymeric materials.

Mg is an essential mineral element for many physiological functions in the human body. Although Mg is the lightest metal, the mechanical strength of the reinforced Mg alloy is comparable with that of aluminum alloy and steel. Ma et al. [89] used magnesium-based alloys as stent materials and analyzed the influence of Mg^2+^ on vascular smooth muscle cells. The results showed that low concentrations (<10 mM) of Mg^2+^ can increase cell adhesion, cell spreading, cell viability, cell proliferation rate, cell migration rate, and actin expression, but high concentrations (40–60 mM) of Mg^2+^ may cause adverse reactions on the cells. Kirkland et al. [90] studied the dissolution rates of different Mg alloys in the simulated body fluid and demonstrated their feasibility for medical implants. The first commercial absorbable metallic stent was the AMS-1 BDS (AMS-1, Biotronik AG, Bülach, Switzerland), made of Mg alloy (Mg > 90%, rare earth metals < 10%) [91]. AMS-1 BDS has shown good mechanical support and degradation performance in the clinical trial [92]. Cao et al. [93] investigated the in vitro corrosion properties of Mg matrix in situ composites, and the results indicated that Mg-10 wt% (weight percent) ZnO composites displayed the lowest corrosion rate. Patil et al. [94] studied the corrosion behavior of Mg samples with self-assembled alkylsilane coatings, and the results showed that the corrosion rate was dramatically reduced.

Zn is a new biomaterial used in biodegradable stent manufacturing due to the fact of its excellent catalytic, structural, and regulatory properties [95]. Zn has good corrosion behavior, which makes it a promising material candidate for biodegradable stents. Bowen et al. [96] proposed Zn as a biodegradable stent material first. Guillory et al. [97] investigated the effect of corrosion characteristics on the long-term inflammatory profile of degradable zinc arterial implants. Drelich et al. [98] conducted a long-term follow up of zinc implants in the murine artery, and the results indicated that Zn stents can be bio-integrated into the arterial environment and safely degrade within 1–2 years. Drelich et al. [99] studied the effect of surface finishing (oxidation, electropolishing, and anodization) on the degradation behavior of Zn stent and found that the oxide film made a great impact on the degradation rate after stent implantation. Jarzębska et al. [100] carried out experiments to investigate the influence of severe plastic deformation on the mechanical properties and microstructure of biodegradable zinc alloy with 1 wt% magnesium and pointed out that this alloy with enforced strength may satisfy the requirements for stent application.

In addition to metal materials, biodegradable polymeric materials have also been widely used in stent applications. Poly-L-lactic acid (PLLA), as the most common polymer material, was first applied as stent material [101]. The initial 6 month clinical results suggested that PLLA biodegradable stents are feasible, safe, and effective. Grabow et al. [102] studied the effect of plasticizer addition on the mechanical properties of PLLA materials, and the results indicated that plasticizer addition increased the elongation at break of PLLA obviously, while it adversely affected the creep behavior of PLLA. Subsequently, other biodegradable polymer materials, including poly(lactide-co-glycolide) (PLGA), polyε-caprolactone PCL, poly-glycolic acid PGA, poly(D-lactide) PDLA, have been explored for stent application [103,104]. The polymeric materials show great advantages in biodegradation properties, biocompatibility, and drug delivery performance. However, the mechanical property of the polymer is insufficient, so further research is required to improve the mechanical properties of the polymer through material processing, precise manufacturing techniques, and post-processing techniques.

## 4. Stent Manufacturing Techniques

### 4.1. Braiding Technique

The braiding technique is to wind a wire around the carrier, and then the wire is braided along the axis of rotation in the prepared track to fabricate the mesh-like stent.

**Figure 1:**
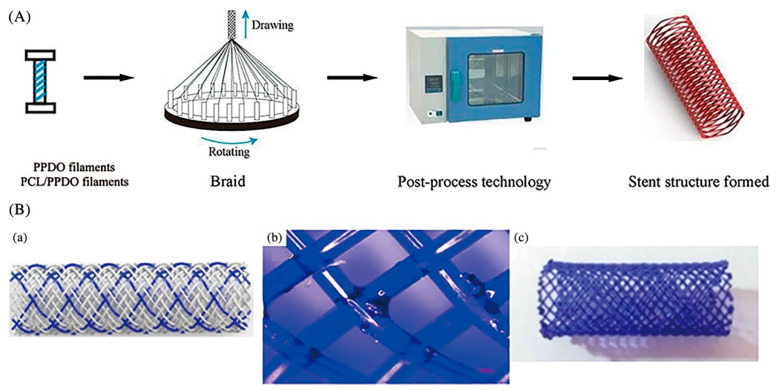
The braiding process of a biodegradable stent (BBS). (**A**) Scheme of stent production. (**B**) (**a**) geometrical model; (**b**) bond at the interlacing point with PCL/PPDO composite filament and PPDO monofilament; (**c**) sample [40], Copyright © 1999–2022 John Wiley & Sons, Inc.

Ueng et al. [39] fabricated vascular stents by braiding stainless-steel fibers combined with nitinol fibers (0.08 mm in diameter). Sun et al. [40] applied the braiding technique for biodegradable stent fabrication by using poly(p-dioxanone) (PPDO) monofilaments and PCL/PPDO composite filaments, and mechanical testing results showed that the mechanical properties of the braided biodegradable stents were comparable to metallic stents including elastic recovery rate, deformation rate, and expansion behavior, as shown in Figure 1.

Overall, the braiding technique is more suitable for the fabrication of compliant shape memory polymeric stents due to the limitations of simple structure and poor radial stiffness.

### 4.2. Micro-Injection Molding

Micro-injection molding is a forming process using molds. Generally, the polymer is heated, melted, and then sent to the mold to form the designed shape after cooling.

Micro-injection molding has many advantages including high processing efficiency, good surface quality, high reproducibility, good material condensation orientation, and good forming consistency [105,106]. Holzer et al. [107] fabricated grooves of 18 nm in width on polymer. Lee et al. [108] combined Moldflow and pressure-driven deformation modeling to optimize the process of micro-injection molding and produced 300 nm grating texture. Stormonth-Darling et al. [109] produced a pillar nanostructure with an ultra-high aspect ratio of up to 20:1.

Huang et al. [41] attempted to fabricate polymeric vascular stents by injection molding for the first time and successfully obtained some patent authorizations. Their achievements bring out the possibility for the batch production of polymeric stents. Li et al. [42] carried out numerical simulations on the micro-injection molding process of polymeric stents by integrating the design of the experiment and the kriging surrogate model, and the results showed that the residual stress and warpage can be significantly reduced. A novel balloon-expandable self-locking poly(e-caprolactone) stent designed by Liu et al. [43] was fabricated by micro-injection molding and spray-coating techniques. The mechanical test results showed that they possess good self-locking characteristics and compression strengths.

Actually, the micro-injection molding method is not widely applied in stent manufacturing. There are still many problems during the micro-injection molding process, including serious material filling and demolding problems, due to the stents’ tiny size and complex structure.

### 4.3. Laser Cutting

**Figure 2:**
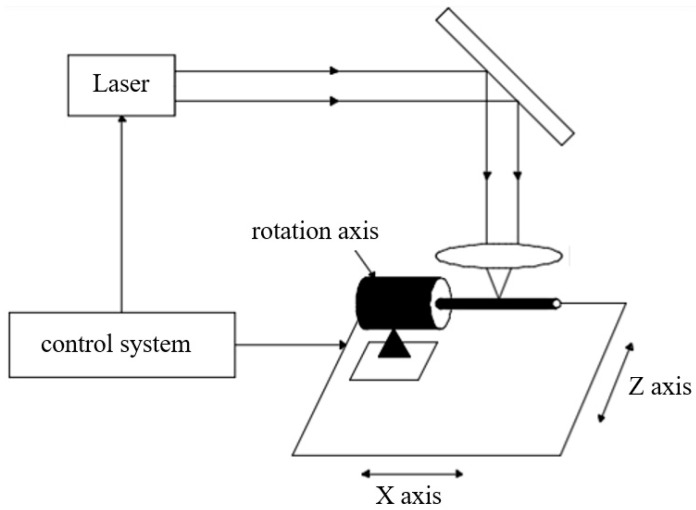
Laser cutting process of a vascular stent.

Laser cutting is the most common method used for vascular stent fabrication, as shown in Figure 2. During the laser cutting process, a high-power laser boom focuses on the tubular material, the material quickly melts, vaporizes, or ablates and then the material is blown away by high-speed airflow.

Momma et al. [44] used the traditional industrial laser to cut slotted tubular coronary stents, and steel stents with high quality were obtained. However, the traditional industrial laser is limited to material types, so that higher precision laser processing techniques are required. Li et al. [45] optimized the femtosecond laser cutting process of NiTi shape memory alloy and fabricated a self-expanding medical micro-device with high accuracy. Raval et al. [46] fabricated a stent with complex geometry based on 316 LVM tubes by a CNC-controlled pulsed neodymium-doped yttrium–aluminum garnet (Nd: YAG) laser. Chen et al. [47] produced a high-quality 316 LVM stainless steel vascular stent by optimizing the laser processing parameters including the lens’ focal length, focus position, pulse frequency, cutting speed, and pulse width. Kathuria et al. [48] applied the short pulse Nd-YAG laser to fabricate a stent with a diameter of 2.0 mm and a length of 20 mm and found that the cutting surface quality can be improved by controlling the heat-affected zone and dross removal process. Erika et al. [49] investigated the formation mechanism of back wall dross and surface roughness during fiber laser micro-cutting of 316 L miniature tubes and fabricated the stents with a surface roughness of less than 1 µm and dross deposits of less than 3.5%. Bear et al. [50] designed and fabricated a novel laser-activated shape memory polymer stent, and the deformation of the fabricated stents was calibrated in a water-filled artery model in vitro. Stepak et al. [51] obtained a PLLA/PLGA stent with a strut width of 300 μm by CO_2_ laser cutting, but the mechanical properties of the stents were severely weakened due to the large areas of the heat-affected zone during stent processing. Demir et al. [52] fabricated an AZ31 magnesium alloy cardiovascular stent with a novel mesh design by laser micromachining, and the obtained stent had a diameter of 2.5 mm and a thickness of 0.2 mm. Liu et al. [53] fabricated NiTi shape memory vascular stents by fiber laser cutting and studied the influence of cutting parameters on cutting quality including the surface roughness, kerf width, heat-affected zone, and dross formation. The results showed that the cutting quality could be improved by optimizing power density along the cutting direction. Guerra et al. [54] machined PCL, PLA, and PCL–PLA tubes to obtain mesh-like stents by fiber laser cutting and analyzed the effect of power, cutting speed, and the number of passes on penetration, precision, and dross. It suggests that fiber laser has great potential in PCL stent machining. Meng et al. [55] designed a fiber laser cutting system for metallic stent fabrication, and the experimental results indicated that the kerf width plays an important role in the machining quality of 316 L stainless-steel stents. Muhammad et al. [56] fabricated nitinol and platinum–iridium alloy vascular stent by picosecond laser micromachining. Although laser cutting is the most widely used method in vascular stent fabrication, there are still many disadvantages. Particularly, large heat-affected zones will appear after laser processing, which lead to a decrease in mechanical properties and biocompatibility for the vascular stent. Therefore, some other stent processing methods are constantly being explored and attempted.

### 4.4. 3D Printing

3D printing, also known as additive manufacturing technology, is a manufacturing process that creates a physical object from a digital model. The technique is realized by adding layer upon layer of material to build up a complete object. 3D printing technology is developing rapidly, and the most widely used 3D printing techniques include selective laser melting (SLM), stereo lithography (SLA), and fused deposition modeling (FDM). 3D printing has many advantages, including flexible processing, various materials, and personalized structure. Therefore, many researchers focus on this method to manufacture vascular stents for personalized patients.

**Figure 3:**
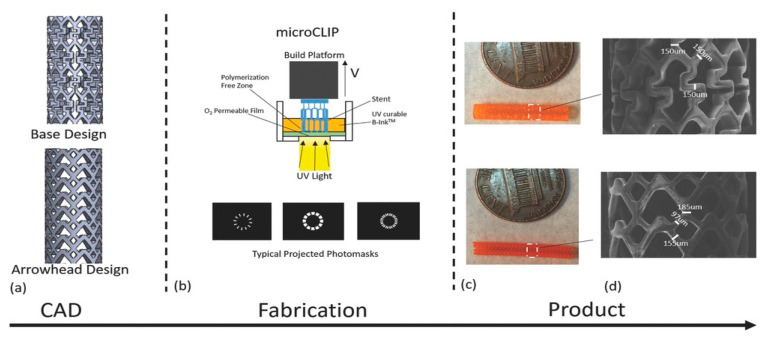
SLA processing of vascular stents. (**a**) CAD images of the initial/primary design (Base Design) and a secondary design (Arrowhead Design). (**b**) Diagram of continuous liquid interface production microstereolithography (microCLIP) with typical projected photomasks of the stent. (**c**) 3D-printed base design (**top**) and arrowhead design (**bottom**) stents. (**d**) Scanning electron microscopy images of the base design (**top**) and arrowhead design (**bottom**) [59], Copyright © 1999–2022 John Wiley & Sons, Inc.

**Figure 4:**
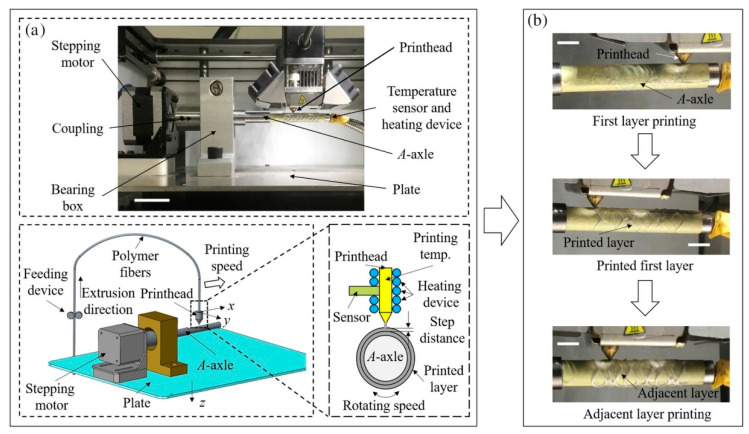
FDM processing of stents. (**a**) 3D printer machine and machine methodology. (**b**) Fabrication process [64], Copyright © 1999–2022 John Wiley & Sons, Inc.

Flege et al. [57] fabricated customized biodegradable vascular PLLA and PCL stents by the SLM technique for the first time and demonstrated that they have good biocompatibility. Finazzi et al. [58] produced a novel cobalt–chromium (CoCr) alloy balloon-expandable stents using an industrial SLM system, and the balloon expansion experiments showed that the stent had good mechanical properties. Van Lith et al. [59] fabricated a novel stent with bioresorption and antioxidant properties using a costumed micro-continuous liquid interface production system (μCLIP). The printed stent had a bridge width of 150 μm and struct thickness of 500 μm and showed good mechanical properties. Ware et al, as shown in Figure 3, optimized the μCLIP process by adjusting the entangled process parameters and applying a novel speed working curve method. They obtained a biodegradable stent with a strut thickness of 150 mm, and the radial stiffness was comparable to nitinol stents. Guerra et al. [61] produced PCL/PLA composite vascular stents by a novel 3D printing technology based on FDM. The 3D printed stents showed excellent properties including dynamic mechanical behavior, expansion behavior, and degradation behavior. Lin et al. [62] fabricated shape memory stents with a negative Poisson ratio structure by 4D printing, and the stent deformation can be validated by increasing temperature. Jia et al. [63] used shape memory PLA to fabricate self-expandable biodegradable vascular stents by 3D printing technology, and the stents had good shape memory function. Zhao et al. [64] developed a novel 3D printing system to improve printing accuracy by integrating a rotating shaft with controllable rotating speed and temperature, and the developed system showed great potential in manufacturing stents with variable structures, as shown in Figure 4.

### 4.5. Other Manufacturing Techniques

In addition to the above methods, other processing techniques, such as micro-electrical discharge machining (μEDM), micro-photochemical etching, magnetron sputtering, micro-precision milling, and some combined processing method, have also been developed for vascular stent manufacturing.

Takahata et al. [110] fabricated stents from 50 μm thick stainless-steel foil by using μEDM technology, and mechanical testing results showed that the fabricated stent had good radial stiffness and bending compliance. Kuribayashi et al. [111] produced a new origami stent graft from Ni-rich TiNi shape memory alloy foil by negative photochemical etching. Lima de Miranda et al. [112] integrated magnetron sputtering, three-dimensional photolithography, and wet etching techniques to fabricate shape memory alloy stents from thin TiNi films. Rumpf et al. [113] used DC magnetron sputtering on NiTi tubes to manufacture stents with a diameter of 400 μm and a wall thickness of 50 μm. Moreover, precision milling has many advantages for stent manufacturing such as fast processing speed and high processing accuracy. Especially, the precision milling technique produces high cutting efficiency and cutting accuracy with the intensive research and rapid development of micro-cutting tools including the tool’s service life and the cutting edge. However, research publications on precision cutting methods for cardiovascular stent manufacturing are rare. Table 1 describes the advantages and disadvantages of common vascular stent processing methods.

## 5. Post-Processing Techniques

### 5.1. Drug Coating

The surface can be coated with a layer for drug delivery after stent processing, which can reduce the complications after stent implantation by controlling the release of drugs and by improving the biocompatibility and long-term efficacy and safety of stents. The rate of drug release is influenced by many factors including the diffusion coefficient, the dissolution coefficient, the rate of drug absorption into the tissue, and the penetration rate of the vessel wall. In order to achieve good therapeutic effects, the drug coating of vascular stents has many requirements. First of all, targeted drugs should be lipophilic to ensure the concentration of drugs in the diseased blood vessels are maintained at a relatively high level. Then, the drugs possess the ability to inhibit the excessive proliferation of smooth muscle cells and neointimal hyperplasia. In addition, drugs need to be able to resist platelet adhesion and eliminate inflammation and thrombosis. However, the existing targeted drugs used in the surface coating of vascular scaffolds cannot fulfill all of the above requirements at the same time. Table 2 summarizes the commonly used drugs in stent coatings according to their structural formula, mode of action, and products.

Yair Levy et al. [65] coated rapamycin on the surface of the metallic stent using a temperature induced crystallization method. The in vitro drug release testing results showed that the drug release rate was more than 70% at 15 days. Wahid Khan et al. [66] investigated the drug release rate of carrier free-rapamycin coated CoCr alloy stents, and the results showed that the drug release rate was fast and highly dependent on the releasing condition. Chen et al. [67] used an ultrasonic spray-coating method to make sirolimus/PLGA coatings on stents and studied the influence of the solvent types, spraying process parameters, and the plasticizer addition on the coating quality. In vitro drug release rates changed with the various ratios of sirolimus/PLGA, but there was no linear interaction between the drug release rate and the ratio of the drug/polymer. Raval et al. [68] modified the conventional airbrush technique to coat Co–Cr L605 metallic stents with the drug sirolimus and biodegradable polymeric-matrix-mixed layers. Drug releasing mechanisms were studied, and the results indicated that the drug release rate was faster in hydrophilic layer coatings compared with the hydrophobic coatings. Petersen et al. [69] produced a polymer coating containing antiproliferative drugs on stents and studied the drug-releasing mechanisms in the internal and external coatings. The results showed that this coating method could inhibit the proliferation of smooth muscle cells, reduce platelet adhesion, and promote endothelial cell growth. Van der Giessen et al. [70] investigated the effects of rapamycin doses on the clinical efficacy of drug-eluting stents, and the results showed that there was no significant difference.

A stent surface with microgrooves can reduce the dose of anti-proliferative drugs and improve the efficiency of drug release. In this way, Firehawk (MicroPort, Shanghai, China) is designed with drug coatings containing the minimum dose of rapamycin clinically. The Jactax stent (Boston Scientific, Marlborough, MA, USA) has 2750 discrete micropores on the outer surface, and a mixture of paclitaxel and PLA (50/50) was added to these micropores [114]. Another coating preparation method is to process the penetrating micropores on the stents. Conor stents (Conor Medsystems, Menlo Park, CA, USA) are designed with drug coatings by this method, and the mixture of PLGA and paclitaxel is placed in each micropore. Although the drug release rate is relatively fast, in vivo tests have shown that the stents had a stronger inhibitory effect against neointimal hyperplasia than the stents with a long-term drug release coating [115]. In the penetrating micropores, anti-proliferative drugs can be loaded on the side of the vessel wall, and pro-endothelialization drugs can be loaded on side of the blood. In this way, the endothelialization of the stent is not delayed, and the probability of complications, such as late thrombosis and in-stent restenosis, can be reduced.

### 5.2. Surface Modification

Surface modification is to treat the stent surface by physical or chemical methods so that the stent has better biocompatibility. Surface modification is conducive to the recovery of damaged blood vessels, improves blood contact characteristics, and enhances the migration, adhesion, and proliferation of endothelial cells. At the same time, it also helps to control the drug release rate of stents.

Meng et al. [71] deposited a chitosan/heparin coating onto vascular stents by layer-by-layer self-assembly method, which promoted the process of reendothelialization and intimal healing after stent implantation. Qiu et al. [72] developed a biodegradable vascular stent with surface modified by 2-N, 6-O-sulfated chitosan. The experimental results showed that the microstructure of the modified stent changed, but the mechanical properties were not significantly influenced. The modified stent had good compatibility with blood and cells and could promote cell proliferation. Hossfeld et al. [73] modified the surface of DESs by hyaluronic acid/chitosan films. The animal experiment results showed that modified stents could reduce the adsorption of blood cells and had a good effect on inhibiting thrombosis. Kim et al. [74] designed a multi-layer stent surface, which is coated by hyaluronic acid micelles, heparin, and poly-L-lysine, and the designed stents can inhibit the proliferation of smooth muscle cells.

### 5.3. Microstructures

Surface microstructures, including microgroove array, micropillar array, microlens array, and surface micropatterning [116,117], have the advantages of reducing resistance, improving lubrication and superhydrophobicity, and improving the physical properties of the material surface. Fabricating microstructures on the surface of stents can improve blood compatibility and blood fluidity after implantation and inhibit the adhesion of macromolecules in the blood.

The surface of metallic stents was treated with parallel grooves and compared with smooth controls by Palmaz et al. [75]. The results found that parallel grooves on the surface significantly increased the migration rate of endothelial cells. Wang et al. [76] produced microstructures with different periods on the surface of metallic vascular stents and loaded the mixture of polymer and drug into the microstructures. The results showed that the microstructure with a period of 10 μm had the best drug adhesion and releasing properties. Aguilar et al. [77] produced micropatterning by ultraviolet laser and femtosecond laser on the biodegradable polymer surface, and the results showed that both laser processing methods had the advantages of high-precision, convenience, flexibility, and no harmful chemical composition for polymeric materials. Ma et al. [78] prepared a multifunctional 3D micro–nano structure by temporally shaped fs laser ablation on the surface of NiTi alloy stent. The biological experiments showed that the structure can effectively inhibit the proliferation of bacteria to form a biofilm and has good antibacterial infection ability. Ding et al. [79] studied the effect of microstructure geometry and size on the biocompatibility of material, and the results showed that 1 μm groove microstructures are good for cell adhesion and proliferation. Moreover, both groove and pillar microstructures can inhibit the growth and expansion of smooth muscle cells.

### 5.4. Other Post-Processing Techniques

In addition to the above commonly used treatment processes, other strategies, such as polishing and oxidation treatment, can also promote the biocompatibility of vascular stents.

Plant et al. [118] treated the surface of NiTi alloy by mechanical polishing and oxidation heating treatment and found that the integrity of human endothelial monolayers on NiTi alloy can be controlled by surface treatment. Hryniewicz et al. [119] improved surface corrosion resistance and fatigue resistance properties of CoCr alloy by electrolytic polishing under a magnetic field. Demir et al. [120] applied the electrochemical polishing technique to improve the surface quality of the CoCr stents and demonstrated that the adhered particles and molten material can be removed by this polishing technique.

## 6. Conclusions

This paper comprehensively analyzed the manufacturing technology of vascular stents and summarized the material selection, precision manufacturing, manufacturing post-processing, and other aspects. These factors have a great influence on the performance of vascular stents. The existing technique for stent fabrication can satisfy basic clinical requirements. However, there are still many limitations and shortcomings of existing stent manufacturing technology, especially for difficult miscellaneous diseases and personalized demands.

Precision milling has great advantages, such as high processing efficiency and high processing accuracy and has great potential in vascular stent manufacturing. Similarly, post-processing techniques, such as complex surface textures, ion sputtering, and biocorrosion, can also be used to improve the surface quality and accuracy of the fabricated vascular stents. Overall, vascular stent manufacturing technology will present multi-process combined and diversified development in the future.

## Figures and Tables

**Table 1:** Comparison of various manufacturing techniques used in vascular stents.

Methods	Advantages	Disadvantages
Braiding technique [39,40]	Easy to process	Limited to simple structure Poor radial stiffness
Micro-injection molding [41,42,43]	High production efficiency Good surface quality High consistency	Difficult to processing
Laser cutting [44,45,46,47,48,49,50,51,52,53,54,55,56]	Good quality High processing accuracy	Heat-affected zone
3D printing [57,58,59,60,61,62,63,64]	Personalized customization High material utilization	Poor accuracy
μEDM Micro-photochemical etching Magnetron sputtering [110,111,112,113]	Burr/dross-free	Limited to specific materials
Micro-precision milling	High production efficiency High processing accuracy	Burrs

**Table 2:** Drugs commonly used in a vascular stent.

Drug	Structural Formula	Mode of Action	Products
Sirolimus	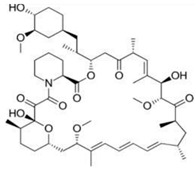	Anti-proliferative, immunosuppressive	Cordis Corporation, Hialeah, FL, USA Abbott Vascular, Temecula, CA, USA Biotronik, Berlin, Germany MicroPort, Shanghai, China
Everolimus	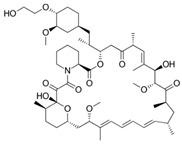	Immunosuppressive	Cordis Corporation, Hialeah, FL, USA Abbott Vascular, Temecula, CA, USA Biotronik, Berlin, Germany MicroPort, Shanghai, China
Paclitaxel	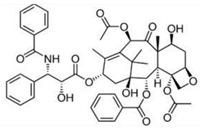	Anti-proliferative agent	Boston Scientific, Marlborough, MA, USA Conor Medsystems, Menlo Park, CA, USA Cook Medical, Bloomington, IN, USA Biotronik, Berlin, Germany Sahajanand Medical, Surat India
Tacrolimus	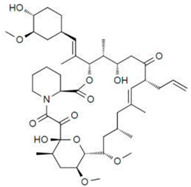	Anti-proliferative, immunosuppressive	Kaneka Corporation, Osaka, Japan Sorin Biomedica, Saluggia, Italy
Zotarolimus	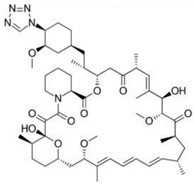	Anti-proliferative, immunosuppressive	Medtronic CardioVascular, Minneapolis, MN, USA
Umirolimus	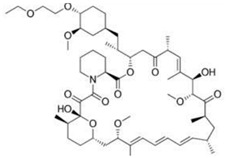	Immunosuppressive	Biosensors Inc., Schenectady, NY, USA Terumo Corporation, Tokyo, Japan Biosensors Europe SA, Morges, Switzerland
